# The authors reply: Letter on: “Fibroblast growth factor 21 controls mitophagy and muscle mass” by Oost et al.

**DOI:** 10.1002/jcsm.12500

**Published:** 2019-11-06

**Authors:** Lynette J. Oost, Marco Sandri, Vanina Romanello

**Affiliations:** ^1^ Venetian Institute of Molecular Medicine Padova Italy; ^2^ Department of Physiology Radboud University Medical Center Nijmegen The Netherlands; ^3^ Department of Biomedical Science University of Padova Padova Italy; ^4^ Myology Center, Department of Biomedical Science University of Padova Padova Italy; ^5^ Department of Medicine McGill University Montreal QC Canada

In response to the letter of Wu *et al*.,^1^ which highlighted some critical biological function of the classical FGF21 signalling in muscle physiology, we would like to clarify some points that should not be misinterpreted. In the last years, our understanding of the physiological role of FGF21 has been dramatically challenged. In skeletal muscle, FGF21 levels are almost undetectable in basal conditions. In contrast, FGF21 is induced in physiological conditions like exercise or during pathophysiological situations such as mitochondrial myopathies or aging, suggesting that muscle‐derived FGF21 has a role in both health and disease.^2^ The beneficial or detrimental effects of FGF21 will result from the combination of several factors including (i) the tissue sources; (ii) the circulating FGF21 half‐life; (iii) the presence of synergizing or antagonizing factors; (iv) the age of the animals/organism (youth and anabolic state versus aged and catabolic state); (v) the FGF21 blood level that, when reaches a certain threshold, can elicit negative effects; and (vi) acute and transient versus chronic and persistent FGF21 secretion. Thus, depending on the combination of these variables, FGF21 can be both a therapeutic agent and a biomarker of disease^2^, ^3^. We agree with Wu *et al*. that the different outcomes complicate the understanding of FGF21 biological role.

The first issue raised in Wu *et al*.^1^ is that the activation of PGC1α, a master regulator of mitochondrial biogenesis, might mediate FGF21‐dependent muscle loss. The authors' hypothesis is supported by the data that acute overexpression of FGF21 in C2C12 myoblasts increases PGC1α, which would lead to an enrichment of smaller fibres due to a glycolytic‐to‐oxidative fibre‐type switch.^4^ However, this assumption is not confirmed by the transgenic overexpression of PGC1α in skeletal muscle, which promotes a metabolic shift but does not alter fibre size in young mice.^5^ Consistently, the cross‐sectional area of myofibers from aged PGC1a transgenic mice is slightly increased when compared with age‐matched controls.^6^ Moreover, muscle wasting is associated with downregulation of PGC1α expression, and the specific maintenance of PGC1α in skeletal muscle during atrophy is sufficient to counteract muscle loss.^5^, ^7^ These protective effects explain why exercise, by increasing PGC1α levels, is beneficial for muscle mass maintenance in several diseases. Finally, the size of Type 1 and 2A fibres from soleus, a PGC1α‐enriched muscle, is bigger than the size of Type 1 and 2A fibres from PGC1 α‐poor muscles like EDL.^8^ Therefore, the data do not support a major involvement of an acute PGC1 α‐mediated metabolic shift as a trigger of muscle atrophy.

In skeletal muscle, FGF21 is produced and secreted in response to mitochondrial stress. The dysfunctional mitochondria activate different pathways that control mitochondrial biogenesis, morphology and dynamics, and mitophagy to restore or improve the quality of the mitochondrial network. In line with this, Wu *et al*. raised a second concern, claiming that acute FGF21 induction in C2C12 myoblasts enhances mitochondrial oxidative function via the activation of mTOR‐Yin Yang1‐PGC1α pathway, as previously reported by Ji *et al*.^9^ However, mitochondrial myopathies which have chronically high FGF21 levels do not show an increase of PGC1α levels.^10^, ^11^ Similarly, during aging, despite the increase of FGF21 in the blood,^12^ the levels of PGC1α are reduced in skeletal muscles of elderly people.^13^ Moreover, a therapeutic strategy to promote mitochondrial biogenesis in patients with mitochondrial myopathy is under clinical trial,^14^ and the transgenic maintenance of PGC1α in skeletal muscle delay the aging process.^6^, ^13^ Therefore, in the context of chronic FGF21 synthesis like aging or mitochondrial myopathies, and in adult myofibers, different mitochondrial quality control pathways, which include but are not limited to mitochondrial biogenesis, are activated. Consistently, we have shown, by gain‐ and loss‐of‐function experiments, *in vivo*, in adult fibres that FGF21 promotes Bnip3‐dependent mitophagy and that this process contributes to muscle mass regulation.^15^ Finally, the signalling pathways that control metabolism might be different in myoblast versus adult myofibers. Thus, FGF21 action on mitochondria is affected by the differentiation state of the cell and by the duration of the stimulus that induces FGF21 expression.

A third point highlighted by Wu *et al*. regards the putative anti‐aging effect of FGF21 co‐receptor β‐Klotho. First, it must be stressed that the anti‐aging actions rely on α‐Klotho and not on β‐Klotho.^16^ α‐Klotho is a membrane‐bound and a circulating protein encoded by a gene in a chromosome different from β‐Klotho. Second, FGF23 binds to α‐Klotho while FGF21 interacts with β‐Klotho. Mice lacking α‐Klotho develop sarcopenia, decreased activity levels, and premature death.^17^, ^18^ In contrast, β‐Klotho inhibition in human myotubes or its specific deletion in skeletal muscle does not significantly alter muscle mass.^19^

Similar to what was reported in the adipose tissue of obese mice,^20^ Wu *et al*. hypothesized that FGF21‐dependent muscle loss might be associated with a reduction of β‐Klotho levels. FGF21‐resistant state was assumed to trigger obesity because obese mice show reduced β‐Klotho levels despite elevated circulating FGF21.^20^ However, this concept has been challenged by the finding that β‐Klotho expression in adipose tissue does not increase FGF21 sensitivity during obesity.^21^ Moreover, during aging, β‐Klotho expression matches with the increase of FGF21, and FGF21 sensitivity is not impaired in the white adipose tissue.^22^ Similarly, both FGF21 and β‐Klotho are induced in atrophic muscles after acute deletion of the mitochondrial fusion gene, OPA1.^12^ We have demonstrated that FGF21 is required for fasting‐dependent muscle mass and force loss.^15^ Importantly, the specific inhibition of FGF21 in skeletal muscle is associated with downregulation of β‐Klotho,^15^ supporting the coordinated regulation of FGF21 and β‐Klotho and their involvement in the control of muscle mass. However, we agree that a better dissection of the FGF21 downstream signalling pathways in adult myofibers and in myogenic stem cells is necessary to explain the biological effects.

In conclusion, the healthy or unhealthy FGF21 action is at least age, time, and dose‐dependent. It should also be considered that FGF21 effect might also be modulated by molecules (cytokines and metabolites) secreted by the different cells present in muscles (e.g. myofibers, satellite cells, fibroblasts, macrophages, smooth muscle, and endothelial cells of the vessels). All these aspects should be considered when drawing conclusions about FGF21 biological role and when extrapolating the findings from cell culture studies to therapeutic interventions for diseases of adult muscles.

## Conflict of Interest

The authors have no conflict of interest to declare.
